# Priority Setting for Universal Health Coverage: We Need to Focus Both on Substance and on Process: Comment on "Priority Setting for Universal Health Coverage: We Need Evidence-Informed Deliberative Processes, not Just More Evidence on Cost-Effectiveness"

**DOI:** 10.15171/ijhpm.2017.06

**Published:** 2017-01-22

**Authors:** Jeremy A. Lauer, Dheepa Rajan, Melanie Y. Bertram

**Affiliations:** ^1^ Economic Analysis and Evaluation, World Health Organization, Geneva, Switzerland.; ^2^ Health Systems Governance, Policy and Aid Effectiveness, World Health Organization, Geneva, Switzerland.

**Keywords:** Priority Setting, Cost-Effectiveness Analysis, Universal Health Coverage (UHC)

## Abstract

In an editorial published in this journal, Baltussen et al argue that information on cost-effectiveness is not sufficient for priority setting for universal health coverage (UHC), a claim which is correct as far as it goes. However, their focus on the procedural legitimacy of ‘micro’ priority setting processes (eg, decisions concerning the reimbursement of specific interventions), and their related assumption that values for priority setting are determined only at this level, leads them to ignore the relevance of higher level, ‘macro’ priority setting processes, for example, consultations held by World Health Organization (WHO) Member States and other global stakeholders that have resulted in widespread consensus on the principles of UHC. Priority setting is not merely about discrete choices, nor should the focus be exclusively (or even mainly) on improving the procedural elements of micro priority setting processes. Systemic activities that shape the health system environment, such as strategic planning, as well as the substantive content of global policy instruments, are critical elements for priority setting for UHC.

While we endorse the call in the editorial by Baltussen et al^1^ for evidence-informed deliberative processes in priority setting for universal health coverage (UHC), we believe that the authors have unnecessarily confused certain questions while leaving others unnecessarily open. To structure our argument, we begin by formulating three specific propositions that we believe are explicit (or implicit) in the editorial:

1. International efforts to assist priority setting in health^2,3^ focus (mainly and unduly) on the provision of cost-effectiveness information (p. 1);
2. Cost-effectiveness information (alone) does not adequately support countries to make choices about priorities (p. 1); and
3. Priority setting is … a “value-laden political process, in which multiple criteria … are important and [in which] stakeholders often justifiably disagree about their relative importance” (p. 1).

Leaving aside here a detailed discussion of DCP3 (which we nevertheless mention in passing later on), and focusing primarily on World Health Organization’s (WHO’s) role, in the first proposition Baltussen et al seem to be restricting their attention only to “WHO-CHOICE”^4^ (which is a programme of the WHO Secretariat initiated precisely for the purpose of making available high-quality, internationally comparable information on cost-effectiveness), while neglecting the broader scope of WHO’s policies, guidance, recommendations, and programmes related to priority setting in health. To the extent that Baltussen et al subscribe to the first proposition, in our view they would be making the error of conflating technical methods (such as WHO-CHOICE), which contribute to the evidence base for priority setting, with the values and principles underpinning a priority setting process.

If on the other hand they do not subscribe to this view, they nevertheless seem to be criticizing a cost-effectiveness programme for producing cost-effectiveness information. The second proposition, therefore, amounts to the assertion that the provision of information on cost-effectiveness (which is an acknowledged international public good) is somehow equivalent to a recommendation that *only* cost-effectiveness information is relevant for priority setting. This proposition, if adhered to by Baltussen et al, would constitute a serious misunderstanding of the aims of WHO-CHOICE.

This brings us to the third proposition, namely that priority setting involves “multiple criteria,” that it is “value laden,” and that “stakeholders often justifiably disagree.” In a related vein, the authors further observe that “society … has a wide range of social values,” and they argue that “the whole of these values should be considered when setting priorities.”

The third proposition appears to us to be the most problematic, since, in this view, the content of the values relevant for priority setting in health appears to be an open question: “stakeholders” with “diverging interests” engage in “pluralistic bargaining” to influence priority setting according to their particular “interests.” Taken to its logical conclusion, this proposition would suggest that – at least in an anarchy of values – legitimacy, reasonableness, and accountability can come only from widespread acceptability of the *procedural* features of social bargaining. While we do not suspect that Baltussen et al actually believe the extreme implications of this third proposition to be true, we nevertheless observe that – in their editorial at least – they have left that possibility far too open.

Although we acknowledge that, at country level, priority setting processes involve a range of stakeholders with different views, with regard to global level policy formulated by WHO’s Member States, it must be recalled that the relevant values enjoy both broad consensus and an international mandate. So, while we agree with the need for an “evidence-informed deliberative process,” such as the authors recommend (especially for generating legitimacy at the relevant decision-making level in a given country), we believe that Baltussen et al have been too agnostic in their editorial about the criteria and values already embodied in UHC (that is, about the substance of UHC). The substance of UHC has been progressively defined and clarified by the Member States of the WHO, as well as by those of the United Nations (UN), in a voluminous series of resolutions, reports, and technical documents.^5-9^ Recently, UHC has been adopted as UN Sustainable Development Goal Target 3.8.^10^ We observe, therefore, in contrast to the apparent agnosticism of Baltussen et al, that these international agreements contain numerous positive assertions concerning the substance and values of priority setting in health.

For example, three main indicators have been identified within the UHC framework:

1. *Coverage:* How many people of those who would stand to benefit are receiving needed health services?
2. *Financial risk protection:* Are people put at risk of impoverishment, or of not accessing health services, because of out-of-pocket payments?
3. *Equity:* Is access to health services (and the financial impact of paying for them) equitably distributed across population groups?^11^

While not exhaustive, these indicators nevertheless constitute a minimum set of criteria that represent the values that Member States of WHO (and of the UN) have endorsed through the organs of governance which have been instituted to determine policy on these matters. In light of the lengthy, participative, transparent, and mandated processes leading to the development of policies on UHC, in our view the above indicators can be held to constitute three ‘essential pillars’ for priority setting in the context of UHC: that is, other values can be added, but it would be a clear distortion of UHC to ignore any of these three. Thus, we conclude that priority setting for UHC does not start with a blank slate, nor is UHC an empty slogan or a catch-all phrase whose content is to be supplied solely through improved (procedural) rules for social bargaining; in fact, the essential content and values required by UHC are articulated in the relevant policies.

To return to what we have identified as the first and second propositions, above, an exclusive focus on WHO-CHOICE (or on DCP3, for that matter) in evaluating international efforts on priority setting is, therefore, incorrect. Furthermore, we note that DCP3 in fact emphasizes methods (called Extended Cost-Effectiveness Analysis) for operationalizing the triple values of health maximization, financial risk protection, and equity for priority setting in health – values corresponding precisely to the UHC indicators mentioned above.

For these same reasons, we would like to reiterate that, at global-policy level and as formulated by the authorized representatives of WHO Member States, there has been an unprecedented amount of agreement on UHC policy, a policy which has itself been shaped through an “evidence-informed deliberative process.” Baltussen et al come closest to acknowledging the centrality of UHC in priority setting when they write,

“In its report *Making fair choices on the path to UHC*, [a consultative group appointed to advise] the WHO recently proposed the use of ‘cost-effectiveness,’ ‘priority to the worse off,’ and ‘financial protection’ as the three most essential criteria for countries to consider when setting priorities^12^ We consider these as ‘core’ criteria, representing social values for which there is broad consensus on their importance and which are of generic relevance across countries, disease areas and health interventions. Their identification can be seen as the product of international learning, particularly in academic circles, on priority setting.”^13^

However, we would add that the criteria referred to in the cited report are not merely the reflections of “academic learning,” nor are they values for which there is simply “broad consensus.” These criteria are in fact those that are embedded *de jure* in the core of UHC policy, a policy which has been adopted by WHO Member States and integrated in the framework of the Sustainable Development Goals.

Furthermore, whereas Baltussen et al stress priority setting in the context of decisions to reimburse specific healthcare interventions, in genuinely organizing health-system priorities around the goals of UHC, some of the required activities thus identified will almost certainly be systemic in nature, such as ‘improving health-system governance,’ ‘ensuring equitable access to quality services,’ ‘separating prescribing from dispensing,’ or ‘setting up a pooled funding mechanism to purchase services.’ None of these system-wide activities are explicitly mentioned by Baltussen et al, although they are arguably instrumental for achieving UHC and might accordingly be given higher priority than decisions, for example, about the introduction of specific services to the benefit package. As a further example, in considering the systemic aspects of priority setting, one should equally take into account other activities not mentioned in the editorial, including health-sector situation analysis, national health strategic-plan development, and health-sector reviews, which comprise an inter-related set of activities that strongly influence the operating environment of discrete decision-making.

This brings us to the claim made by Baltussen et al, to the effect that evidence-informed, deliberative processes in support of priority setting have been mainly “theoretical.” In fact, a substantial portion of the WHO Secretariat’s ongoing work on priority setting is organized around routine strategic-planning activities such as those just mentioned. In this context, country actors and stakeholders are already convening consultative groups for the sake of obtaining consensus on issues of concern. National health-strategy development, health-sector mid-term reviews, and joint annual health-sector reviews are being routinely conducted, and arguably constitute “evidence-informed deliberative processes” of just the kind called for in the editorial. Supporting Member States to manage effectively such routine, regular, and periodic ‘priority setting processes’ (albeit by another name) is an ongoing area of activity for the WHO Secretariat, although we acknowledge that such activities could be further strengthened and supported.

Finally, Baltussen et al highlight the role of health technology assessment (HTA) agencies in priority setting for discrete choices, such as for reimbursement via inclusion in a benefit package. In the light of recent policies established by WHO Member States,^14^ HTA is becoming a fast growing area of work within the WHO Secretariat. In WHO’s conception of HTA, a conception informed by global learning drawn from a variety of approaches, HTA brings together the work of a multi-disciplinary group of experts, including health economists, legal advisors, pharmacists, epidemiologists, ethicists, medical engineers and health systems governance specialists. This responds, at least in part, to another important question left open by Baltussen et al: *who* should be invited to the deliberative dialogue?

In the relevant WHO policies, HTA is not viewed as a technocratic solution to decision-making, nor as one focused on cost-effectiveness analysis, but rather as a transparent and fair evidence-informed decision-making process underpinned by a strong legal framework. With such a vision, provided the content of UHC policy is understood to be at the forefront, and the systemic elements of priority setting receive appropriate emphasis, we believe we can unreservedly find common cause with the proposals of Baltussen et al.

## Ethical issues

Not applicable.

## Competing interests

Authors declare that they have no competing interests.

## Authors’ contributions

JAL drafted the text. DR and MYB revised the text. All contributed to the conception, design, and interpretation; all approved the final version.

## Authors’ affiliations

^1^Economic Analysis and Evaluation, World Health Organization, Geneva, Switzerland. ^2^Health Systems Governance, Policy and Aid Effectiveness, World Health Organization, Geneva, Switzerland.
